# ESPL1 Is a Novel Prognostic Biomarker Associated With the Malignant Features of Glioma

**DOI:** 10.3389/fgene.2021.666106

**Published:** 2021-08-26

**Authors:** Zhendong Liu, Xiaoyu Lian, Xiuru Zhang, Yongjie Zhu, Wang Zhang, Jialin Wang, Hongbo Wang, Binfeng Liu, Zhishuai Ren, Mengjun Zhang, Mingyang Liu, Yanzheng Gao

**Affiliations:** ^1^Department of Surgery of Spine and Spinal Cord, Henan Provincial People’s Hospital, Henan International Joint Laboratory of Intelligentized Orthopedics Innovation and Transformation, Henan Key Laboratory for Intelligent Precision Orthopedics, People’s Hospital of Zhengzhou University, People’s Hospital of Henan University, Zhengzhou, China; ^2^Department of Surgery of Spine and Spinal Cord, Microbiome Laboratory, Henan Provincial People’s Hospital, People’s Hospital of Zhengzhou University, People’s Hospital of Henan University, Zhengzhou, China; ^3^Zhengzhou University People’s Hospital, Henan Provincial People’s Hospital, Zhengzhou, China; ^4^Henan University People’s Hospital, Henan Provincial People’s Hospital, Zhengzhou, China; ^5^Department of Neurosurgery, The First Affiliated Hospital of Harbin Medical University, Harbin, China; ^6^Harbin Medical University Cancer Hospital, Heilongjiang Provincial Cancer Hospital, Harbin, China

**Keywords:** ESPL1, glioma, CGGA, TCGA, prognosis, biomarker

## Abstract

Research has confirmed that extra spindle pole bodies-like 1 (ESPL1), an etiological factor, promotes the malignant progression of cancers. However, the relationship between ESPL1 and glioma has not yet been demonstrated. The purpose of this study was to reveal the potential mechanisms of ESPL1-mediated malignant glioma progression. Gene expression data and detailed clinical information of glioma cases were obtained from multiple public databases. Subsequently, a series of bioinformatics analyses were used to elucidate the effects of ESPL1 on glioma. The results demonstrated that the mRNA and protein levels of ESPL1 in glioma were higher than those in normal brain tissues. In addition, ESPL1 expression was considerably associated with the clinical and pathological features of gliomas, such as World Health Organization grade, histology, and 1p19q co-deletion status. Importantly, ESPL1 reduced the overall survival (OS) of glioma patients and had prognostic value for gliomas. Gene set enrichment analysis (GSEA) indirectly revealed that ESPL1 regulates the activation of cancer-related pathways, such as the cell cycle and base excision repair pathways. In addition, we used the Connectivity Map (CMap) database to screen three molecular drugs that inhibit ESPL1: thioguanosine, antimycin A, and zidovudine. Finally, reverse transcription-quantitative polymerase chain reaction (RT-qPCR) was used to detect the expression levels of ESPL1 in glioma cell lines. This study plays an important role in revealing the etiology of glioma by revealing the function of ESPL1, providing a potential molecular marker for the diagnosis and treatment of glioma, especially low-grade glioma.

## Introduction

Glioma is one of the most prevalent and tricky malignant intracranial tumors due to its infiltrative growth, high degree of malignancy, and unfavorable prognosis (Cordier et al., 2016). According to the 2016 World Health Organization (WHO) guidelines, gliomas are further classified into grades I–IV, of which grades I and II are low-grade gliomas and grades III and IV (glioblastoma, GBM) are high-grade gliomas (Wesseling and Capper, 2018). GBM is the most malignant type; the median survival time is still less than 2 years after maximum resection combined with radiotherapy and chemotherapy, with a 5-year survival rate of only 9.8% (Stupp et al., 2005, 2009). The prognosis of grades II and III gliomas was improved but was still not optimistic at 2 and 2–5 years, respectively (Bell et al., 2015). Although, worldwide, researchers have made significant efforts to facilitate early diagnosis and ensure comprehensive treatment of gliomas, patient prognosis is still not ideal due to its malignant biological characteristics, causing affliction to the families of the patients as well as a huge medical burden to society. One of the reasons for this situation might be the lack of reliable and effective biomarkers for early diagnosis and targeted treatment.

Extra spindle pole bodies-like 1 (ESPL1), a cysteine endopeptidase, plays a vital role in the stable binding between sister chromatids before anaphase and their timely separation during anaphase, which is the key to chromosome inheritance (Chestukhin et al., 2003; Schöckel et al., 2011). When ESPL1 is overactivated, it acts as an oncogene, making cells susceptible to aneuploidy induced by chromosomal mismatch as well as vulnerable to DNA damage and loss of key tumor suppressor gene sites associated with tumorigenesis and disease progression (Pati, 2008; Mukherjee et al., 2014). For example, the overexpression of separase in the mammary glands of mouse mammary tumor virus (MMTV)-ESPL1 mice leads to the occurrence of highly aneuploid breast cancer, which has a high degree of chromosomal instability and an invasive disease phenotype. In addition, Liu J. et al. (2020) showed that the abnormal expression of ESPL1 in endometrial cancer (EC) cells facilitates metastasis and invasion, thereby leading to poor prognosis of EC. ESPL1 also participates in the occurrence and development of other human cancers and is associated with reduced patient survival (Meyer et al., 2009). However, the regulatory mechanisms of ESPL1 in gliomas has not yet been studied. Based on the role of ESPL1 in other tumors, we speculate that ESPL1 might be associated with the clinical features and survival prognosis of glioma patients.

The major aim of this study was to evaluate the expression level, prognostic value, and biological function of ESPL1 based on glioma tissue samples from multiple databases. High expression of ESPL1 was observed at both the mRNA and protein levels by reverse transcription-quantitative polymerase chain reaction (RT-qPCR) and immunohistochemistry (IHC). Our results demonstrated that upregulation of ESPL1 is associated with poor prognosis in glioma patients. Therefore, it is reasonable to speculate that ESPL1 may represent a novel and reliable biomarker for glioma and may aid in the development of individualized treatment strategies.

## Materials and Methods

### Data Collection

Gene expression profiling interactive analysis (GEPIA)^1^ is a convenient and intuitive online public database established by Peking University (Tang et al., 2017). A variety of human tumor and corresponding normal tissue samples are freely available on the website. The database was used to detect the expression levels of ESPL1 in various tumors. The difference in target gene expression in tumor tissues can be obtained by inputting the target gene on the official website. In addition, we downloaded the GSE2223 dataset based on the GPL1833 platform, and the GSE4290 and GSE50161 datasets based on the GPL570 platform from Gene Expression Omnibus (GEO)^2^ (Barrett et al., 2013). GSE2223 contains 50 glioma and 4 normal tissue samples; GSE50161 contains 34 glioma and 13 normal tissue samples; and GSE4290 contains 77 glioma and 23 normal tissue samples. Three different datasets obtained from the GEO database were used to analyze the changes in ESPL1 expression levels in glioma and control brain tissues. These operations were performed using the limma package in R software according to a cut-off standard (*p* < 0.05, logFC > 1) to complete the differential expression of ESPL1 in glioma and control brain tissues.

The Chinese Glioma Genome Atlas (CGGA)^3^ is a public database that contains various types of high-throughput data and corresponding clinical information. By excluding data with incomplete clinical information, we obtained an RNA-seq dataset containing 748 glioma samples and gene microarray data containing 268 glioma samples. The Cancer Genome Atlas (TCGA)^4^ is a credible database that primarily stores several human malignant tumors (Tomczak et al., 2015). We also searched for and obtained mRNA sequencing and clinical information of 653 human gliomas from the TCGA RNA-seq dataset. Supplementary Tables 1–3 provide clinical information of the patients corresponding to the three CGGA RNA-seq, CGGA microarray, and TCGA RNA-seq datasets. The above three original datasets contain detailed data on clinical–molecular characteristics, survival time, and status of glioma patients; thus, they were used to analyze the impact of ESPL1 expression changes on prognosis, clinical–molecular characteristics, and diagnostic value of glioma patients. All these datasets were divided into high- and low-expression groups according to the median expression level of ESPL1 in all samples for subsequent analysis. Statistical significance set at *p* < 0.05 was considered meaningful.

The Human Protein Atlas (HPA)^5^ is a comprehensive and diverse online data platform that contains information about human RNA and protein expression in various cancers (Uhlén et al., 2015; Thul et al., 2017; Uhlen et al., 2017; Thul and Lindskog, 2018). In this study, to detect changes in the expression level of ESPL1 protein in brain glioma tissue samples, we loaded ESPL1 into the database webpage to obtain its expression levels in normal brain, low-grade glioma, and high-grade glioma tissue samples. Therefore, we only observed changes in the ESPL1 protein levels among the groups.

### GSEA

Gene set enrichment analysis (GSEA) is a tool used to predict the function of target genes (Subramanian et al., 2005). We calibrated and normalized the CGGA RNA-seq, CGGA microarray, and TCGA RNA-seq datasets using limma software packages. According to the expression levels of ESPL1, it was divided into high and low expression groups. GSEA 4.0.2 jar software was used to explore the cell signaling pathways of ESPL1 in glioma patients. The number of gene permutations was set to 1000; “C2.cp.kegg.v7.4.symbols.gmt[curated]” was selected as the gene set database. The high expression group of ESPL1 was compared with the low expression group according to the cut-off standard. Values of *p* < 0.05 and FDR < 0.25 were regarded as statistically significant. Finally, the consistent results of the independent datasets are presented in the experimental results section.

### Connectivity Map Predicts Potential Therapeutic Drugs

Connectivity Map (CMap) is a drug research and development system founded by Harvard University and is a common tool for discovering the potential therapeutic effects of drugs (Lamb, 2007). In this study, we used the R language to screen for genes with co-expression relationships with ESPL1. We then selected 20 genes (10 positive and 10 negative) and uploaded them to the official website of CMap for analysis to obtain the corresponding small-molecule compounds. The obtained small-molecule drugs are regarded as valuable drugs according to *p* < 0.001 and enrichment < −0.75, which are presented in the Experimental Results section; the chemical structure formula of the final small-molecule drug as well as its 3D structure were obtained from the PubChem database.^6^

### Cell Culture and Reverse Transcription Quantitative Polymerase Chain Reaction Analysis

Human glioma cell lines (LN229, T98, and A172) and human-derived astrocytes (HA) were purchased from the Cell Bank of the Chinese Academy of Sciences (Shanghai, China). All cells were grown in incubators at 37°C and 5% carbon dioxide and were cultured in DMEM (HyClone, United States) supplemented with 10% FBS (Thermo Fisher Scientific, United States). To examine the expression levels of the three glioma cell lines (LN229, T98, and A172) and HA in ESPL1, total RNA was extracted from LN229, T98, A172, and HA cells using Tri-Reagent (Sigma, United States). Total RNA quality and quantity were determined using a NanoDrop One spectrophotometer (Thermo Fisher Scientific, United States), measuring 260/280 nm absorbance values. Subsequently, the cDNA was reverse transcribed from total RNA using the Transcriptor First Strand cDNA Synthesis kit (Novoprotein Scientific Inc., Shanghai, China). RT-qPCR was performed according to the guidelines for the FastStart Universal SYBR Green Master (ROX) (Novoprotein Scientific Inc., Shanghai, China). The results were quantified using QuantStudio software (Thermo Fisher Scientific, United States), following the manufacturer’s instructions. GADPH was used as an internal reference. The primer sequences used in this study are listed in Table 1. Relative expression levels were determined using the 2^–ΔΔ*Ct*^ method. The expression level of ESPL1 was detected using the “2^–ΔΔ*CT*^” method. Statistical differences were analyzed by unpaired *t*-test; values of *p* < 0.05, were considered statistically significant.

**TABLE 1:** Sequences of primers used for qRT-PCR analysis.

**Gene**	**Primer sequence (5′-3′)**
ESPL1-F	GCCCTAAAACTTACAACAAA
ESPL1-R	AGACTCAAGCAAGAACAGAA
GAPDH-F	CAAGGTCATCCATGACAACTTTG
GAPDH-R	GTCCACCACCCTGTTGCTGTAG

*F, forward; R, reverse.*

### Statistical Analysis

R (v.3.6.1) was used for statistical analysis. Cox regression was used to analyze the relationship between ESPL1 expression and the prognosis of glioma patients; the Kaplan–Meier method was used to create survival curves. Finally, the Wilcox or Kreskas test was utilized to explore the relationship between clinical molecular characteristics and ESPL1 expression in glioma patients. Differences were considered statistically significant at ^∗^*p* < 0.05 or ^∗∗^*p* < 0.01.

## Results

### ESPL1 Is Highly Expressed in Glioma at Different Levels

**FIGURE 1:**
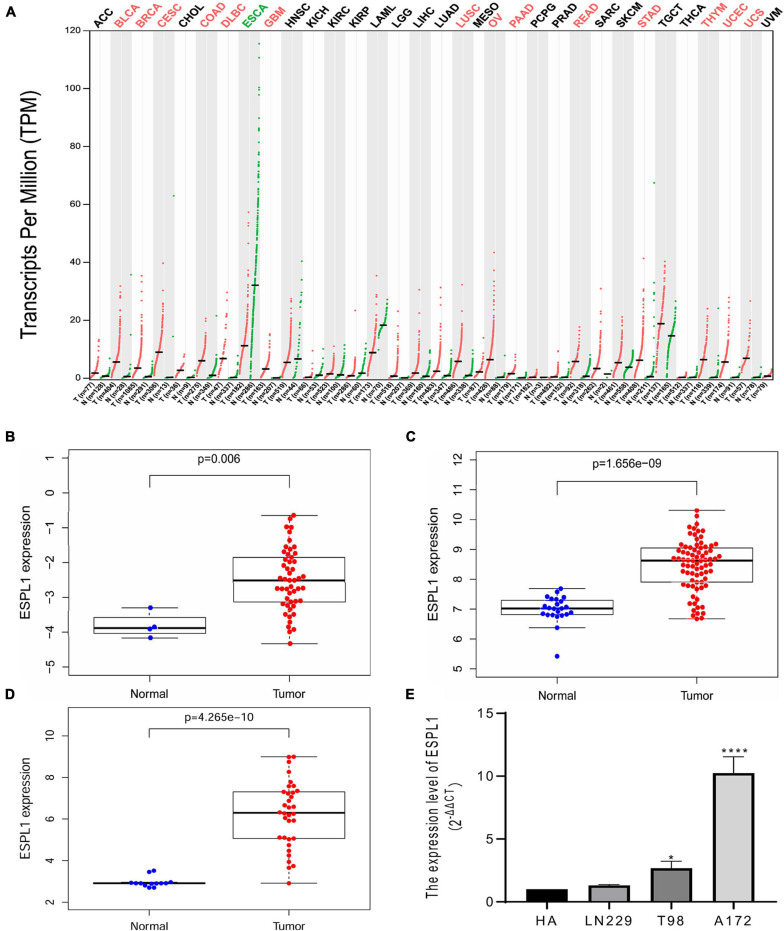
Extra spindle pole bodies-like 1 expression in gliomas. **(A)** The expression of ESPL1 in a variety of malignant tumors. Red indicates that ESPL1 is higher than the corresponding normal control group, while green indicates that ESPL1 is highly expressed in the tumor. **(B)** ESPL1 expression in 50 gliomas and 4 normal brain tissues in GSE2223. The expression of ESPL1 in gliomas was significantly increased. **(C)** In the GSE4290 data, the expression of ESPL1 in 77 gliomas and 23 normal brain tissues was compared, and the expression of ESPL1 in gliomas increased. **(D)** Comparing 34 kinds of glioma tissue specimens with 13 normal brain tissue specimens in the GSE50161 data set, the expression level of ESPL1 in glioma tissue was significantly increased. **(E)** RT-qPCR experiments show that the expression level of ESPL1 in glioma cell lines (LN229, T98, and A172) is higher than that in human astrocytes. *p < 0.05 and ****p < 0.0001.

Extra spindle pole bodies-like 1 expression in various tumors and matched normal tissues was assessed using the GEPIA online tool (Figure 1A); we observed that ESPL1 was abnormally highly expressed in a variety of malignant tumor tissues, including GBM, while the ESPL1 expression level of esophageal carcinoma (ESCA) was lower than that in normal tissues. Thereafter, to understand the changes in ESPL1 expression in glioma tissues at a deeper level, we performed analysis on three glioma-related GSE datasets (GSE2223, GSE4290, GSE50161) from the GEO database, including 40 normal brain and 161 glioma samples. As shown in Figures 1B–D, in these three datasets, the expression levels of ESPL1 in glioma tissues were significantly higher than those in corresponding normal tissues. To validate the above results, we further assessed the expression levels of ESPL1 in three glioma cell lines (T98, U251, and LN229) and in human astrocytes (HA) by RT-qPCR. The results revealed that ESPL1 was markedly overexpressed in glioma cell lines compared to that in HAs (Figure 1E).

### Overexpression of ESPL1 Leads to Poor Overall Survival in Glioma Patients

**FIGURE 2:**
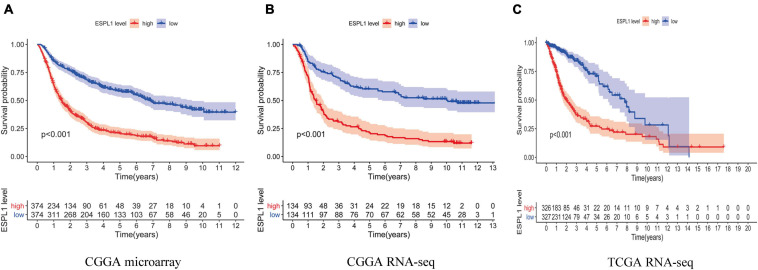
The relationship between the difference of the expression level of ESPL1 and overall survival (OS). The red curve in the figure represents the ESPL1 high expression group, and the blue curve represents the ESPL1 low expression group. **(A)** CGGA RNA-seq dataset. **(B)** CGGA microarray dataset. **(C)** TCGA RNA-seq dataset.

Next, to further examine the effects of abnormally high expression of ESPL1 on the prognosis of glioma, we analyzed three data cohorts: CGGA RNA-seq, CGGA microarray, and TCGA RNA-seq and created survival curves. As indicated in Figures 2A–C, high expression of ESPL1 in the three data cohorts consistently conveyed a significant reduction in patient overall survival (OS) (*p* < 0.001). Because the prognosis of patients with high-grade gliomas and low-grade gliomas is significantly different, the tissue samples were further divided into high- and low-grade gliomas for KM survival analysis to explore the impact of ESPL1 on the prognosis of patients with different grades. The results showed that the change in ESPL1 expression level had no significant difference in the prognosis of high-grade glioma in three independent datasets (Supplementary Figures 1–3B). However, for the prognosis of low-grade gliomas, the increased expression of ESPL1 can indeed reduce the OS time of patients (Supplementary Figures 1–3A). The 2016 WHO grading standard for gliomas also included the molecular characteristics of gliomas in the classification of patients. Therefore, we divided patients into molecular groups to detect the impact of ESPL1 on the prognosis of patients between different molecular categories. The results showed that the increased expression of ESPL1 could significantly reduce the survival time of patients, whether in the IDH mutation group or wild-type group and whether accompanied by 1p19q codeletion or not (Supplementary Figures 1C–E, 2C,D). Although the abovementioned results were obtained from a large sample of 1669 gliomas in 3 data cohorts, whether high expression of ESPL1 represents an independent risk factor for glioma remains to be verified.

### ESPL1 Represents an Independent Risk Factor in Glioma Patients

**FIGURE 3:**
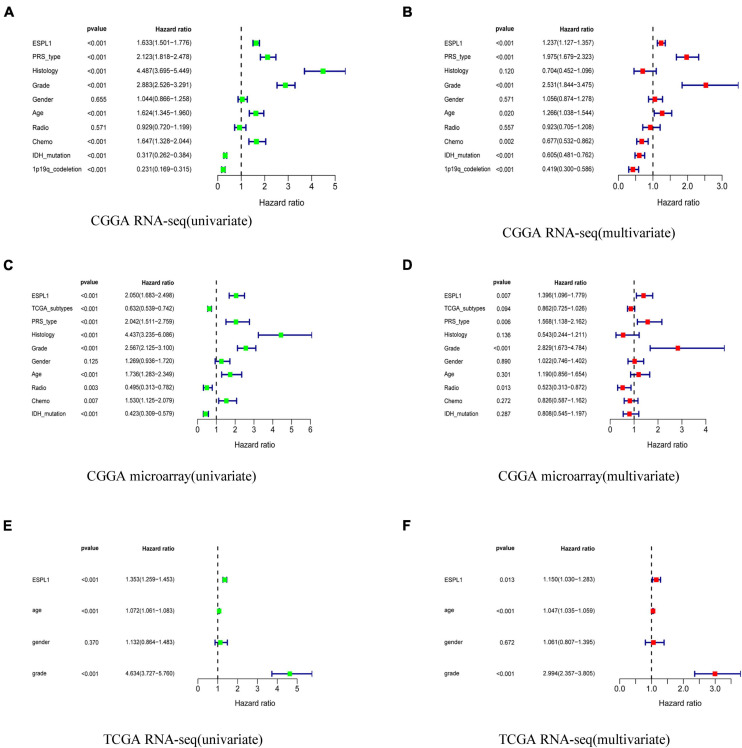
Univariate analysis and multivariate analysis with Cox regression model. **(A)** Univariate analysis of CGGA RNA-seq database. **(B)** Multivariate analysis of CGGA RNA-seq database. **(C)** Univariate analysis of CGGA microarray database. **(D)** Multivariate analysis of CGGA microarray database. **(E)** Univariate analysis of TCGA RNA-seq database. **(F)** Multivariate analysis of TCGA RNA-seq database.

To explore whether ESPL1 represents an independent risk factor for poor prognosis in gliomas, univariate, and multivariate Cox analyses were performed to verify the relationship between high expression of ESPL1 and the prognosis of gliomas. As shown in Figure 3, univariate Cox analysis demonstrated that increased ESPL1 expression in glioma was closely related to poor prognosis in CGGA RNA-seq (HR = 1.633), CGGA microarray (HR = 2.050), and TCGA RNA-seq (HR = 1.353). High-grade CGGA RNA-seq (HR = 2.883), CGGA microarray (HR = 2.567), and TCGA RNA-seq (HR = 4.634), in older patients in CGGA RNA-seq (HR = 1.624), CGGA microarray (HR = 1.736), and TCGA RNA-seq (HR = 1.072), and in the PRS type in CGGA RNA-seq (HR = 2.123) and CGGA microarray (HR = 2.042) (Figures 3A,C,E). At the same time, multivariate Cox analysis revealed that increased expression levels of ESPL1 represent a risk factor in CGGA RNA-seq (HR = 1.237), CGGA microarray (HR = 1.396), and TCGA RNA-seq (HR = 1.103), in older patients in CGGA RNA-seq (HR = 1.266) and TCGA RNA-seq (HR = 1.047), and in PRS type in CGGA RNA-seq (HR = 1.975) and CGGA microarray (HR = 1.568) (Figures 3B,D,F). Consistently, the above results demonstrated that ESPL1 can be regarded as an independent risk factor that conveys an unsatisfactory clinical prognosis.

### The Clinical Diagnostic Value of ESPL1

**FIGURE 4:**
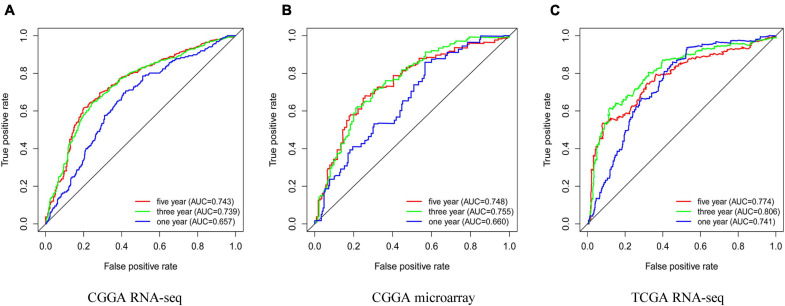
Prognostic factors and risk assessment of glioma and the diagnostic value of ESPL1. **(A)** The receiver operating characteristic (ROC) curve in CGGA sequence. **(B)** ROC curve in CGGA microarray. **(C)** ROC curve in TCGA RNA-seq.

To determine whether high expression of ESPL1 has clinical diagnostic value for the prognosis of glioma, we used Cox regression and Kaplan–Meier methods to draw ROC curves of the CGGA RNA-seq, CGGA microarray, and TCGA RNA-seq cohorts (Figures 4A–C). In these three databases, the area under the curve (AUC) of 3- and 5-years were all greater than 0.7, indicating that ESPL1 has an appropriate diagnostic value. However, in the 1-year survival curve, except for the AUC of TCGA RNA-seq which was 0.741, the AUCs of CGGA RNA-seq and CGGA microarray were all less than 0.7. In addition, in the grading of gliomas, these independent datasets consistently showed that the expression level of ESPL1 has good diagnostic value for the prognosis of low-grade gliomas (Supplementary Figures 4–6A). However, in high-grade gliomas, only the TCGA RNA-seq dataset suggested a good diagnostic value for the prognosis of patients (Supplementary Figures 4–6B). It is worth noting that the expression level of ESPL1 has good diagnostic value among various molecular subtypes in the molecular typing of gliomas (Supplementary Figures 4C–E, 5C,D). In summary, these results indicate that ESPL1 has diagnostic significance for patients with glioma, especially for low-grade gliomas.

### Relationship Between ESPL1 Expression and Clinical Characteristics in Glioma Patients

**FIGURE 5:**
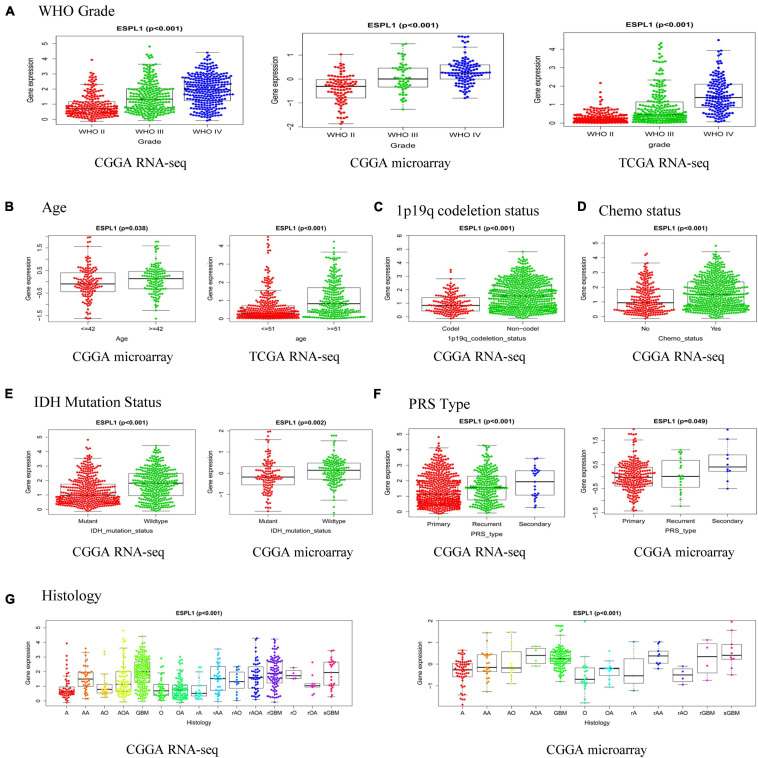
Relationship between the expression of ESPL1 in glioma and clinicopathological characteristics in the CGGA RNA-seq, CGGA microarray, and TCGA RNA-seq datasets. **(A)** Grade. **(B)** Age. **(C)** 1p19q_codeletion status. **(D)** Chemotherapy status. **(E)** IDH mutation status. **(F)** PRS type. **(G)** Histology (A, astrocytoma; AA, anaplastic astrocytoma; AO, anaplastic oligodendroglioma; AOA, anaplastic oligoastrocytoma; GBM, glioblastoma; O, oligodendroglioma; OA, oligoastrocytoma; rA, relapse astrocytoma; rAA, relapse anaplastic astrocytoma; rAO, relapse anaplastic oligodendroglioma; rAOA, relapse anaplastic oligoastrocytoma; rGBM, relapse oligodendroglioma; rO, relapse oligodendroglioma; rOA, relapse oligoastrocytoma; sGBM, secondary relapse oligodendroglioma).

To further investigate the relationship between ESPL1 and the clinical features of glioma patients, we used R software to analyze the three databases in detail. As presented in Figure 5A, high expression of ESPL1 was positively correlated with the WHO grade in the CGGA RNA-seq, CGGA microarray, and TCGA RNA-seq databases (*p* < 0.001). In the CGGA microarray and TCGA RNA-seq, the expression levels of ESPL1 were significantly associated with age (Figure 5B). In CGGA RNA-seq, expression levels of ESPL1 were significantly correlated with 1p19q co-deletion and chemotherapy status (Figures 5C,D, *p* < 0.001). In the two CGGA datasets, expression of ESPL1 was closely correlated with IDH mutation, PRS type, and histology (Figures 5E–G). These results demonstrate that the expression levels of ESPL1 are significantly related to diverse clinical characteristics in glioma patients.

### GSEA Identifies ESPL1-Related Signaling Pathways

**FIGURE 6:**
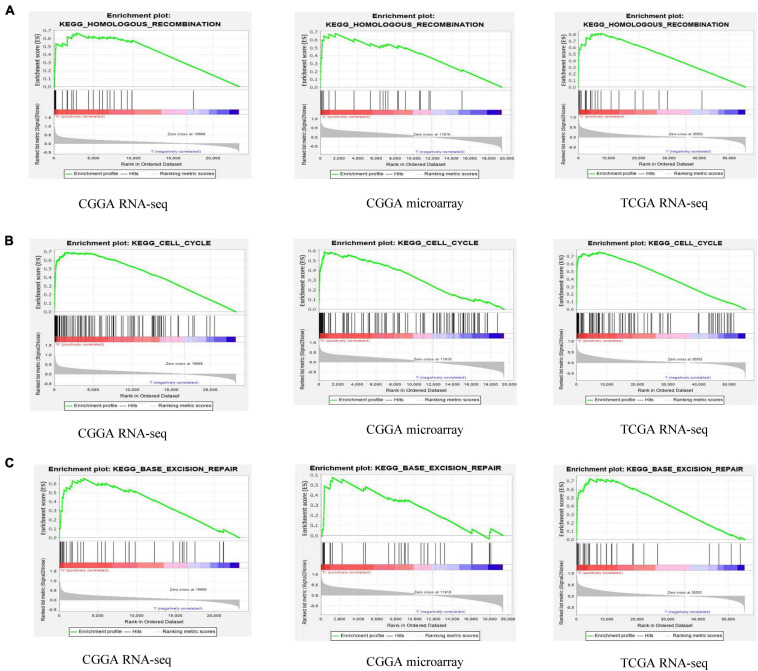
Gene set enrichment analysis (GSEA) of ESPL1 in CGGA RNA-seq, CGGA microarray, and TCGA RNA-seq databases. The results of the three databases show that ESPL1 is associated with three pathways **(A–C)** homologous recombination, cell cycle, and base excision repair.

These results suggest that ESPL1 plays an important role in the pathophysiology of glioma, but the underlying mechanism remains unclear. Therefore, we conducted GSEA analysis to determine whether ESPL1 is involved in tumor-related signaling pathways. The results demonstrated that homologous recombination, cell cycle, and base excision repair were differentially enriched with a high ESPL1 expression phenotype (Table 2 and Figure 6).

**TABLE 2:** The gene set enriches the high ESPL1 expression phenotype.

	**CGGA RNA-seq**			**CGGA microarray**			**TCGA RNA-seq**		
**Gene set name**	**NES**	**NOM *p*-value**	**NOM *q*-value**	**NES**	**NOM *p*-value**	**NOM *q*-value**	**NES**	**NOM *p*-value**	**NOM *q*-value**
Homologous recombination	1.745	0.009	0.116	1.760	0.011	0.382	1.984	0	0.006
Cell cycle	1.964	0	0.031	1.795	0.014	0.513	2.225	0	0.001
Base excision repair	1.840	0	0.082	1.645	0.045	0.550	1.956	0	0.008

*NES, normalized enrichment score; NOM, nominal. Gene sets with NOM *p*-value < 0.05 was considered as significantly enriched.*

### Immunohistochemistry of ESPL1

**FIGURE 7:**
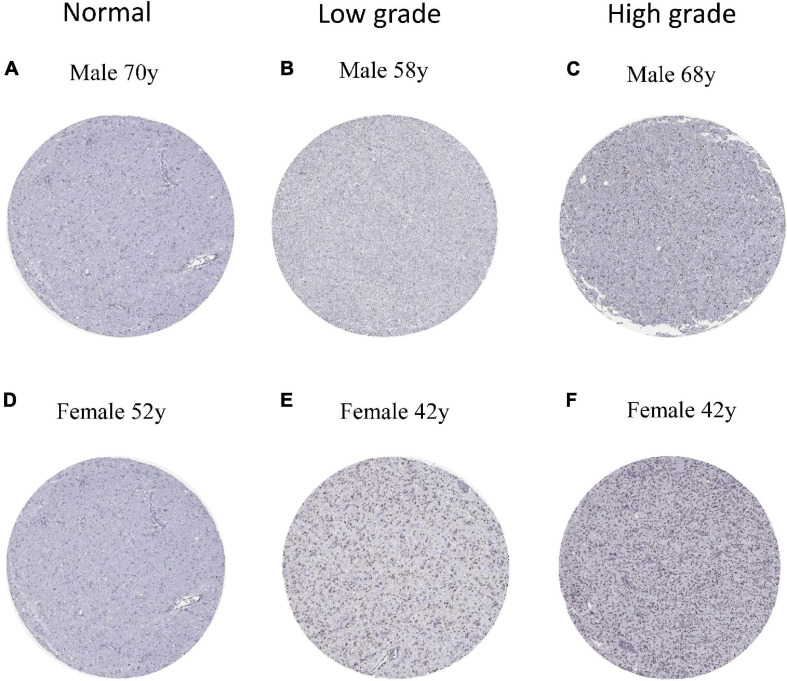
Protein levels of ESPL1 in normal and glioma tissues by immunohistochemistry based on the Human Protein Atlas (staining: medium; intensity: moderate; quantity: >75%). **(A,D)** The expression of ESPL1 in normal brain tissues of men and women. **(B,E)** The expression status of ESPL1 in low-grade gliomas in men and women. **(C,F)** The expression status of ESPL1 in high-grade gliomas in men and women.

To verify the expression of ESPL1 in normal brain and glioma tissues at the protein level, we downloaded six immunohistochemical slices from the HPA^7^ (two normal, two low-grade, and two high-grade), which were stained with HPA073188 (Figures 7A–F). Results showed that ESPL1 protein expression levels in glioma tissue samples were significantly higher than those in normal brains. Furthermore, there was a direct relationship between higher glioma grade and higher expression levels of ESPL1.

### Potential Drugs for the Treatment of Glioma Based on CMap Analysis

**FIGURE 8:**
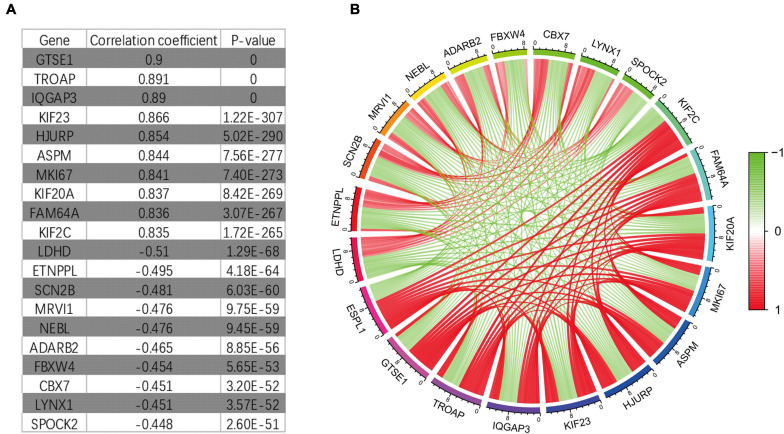
It co-expresses the analysis results. **(A)** Some genes that have synergistic and antagonistic effects with ESPL1, including their names, correlation coefficient values, and *p*-values. **(B)** The expression relationship diagram of genes related to ESPL1.

**FIGURE 9:**
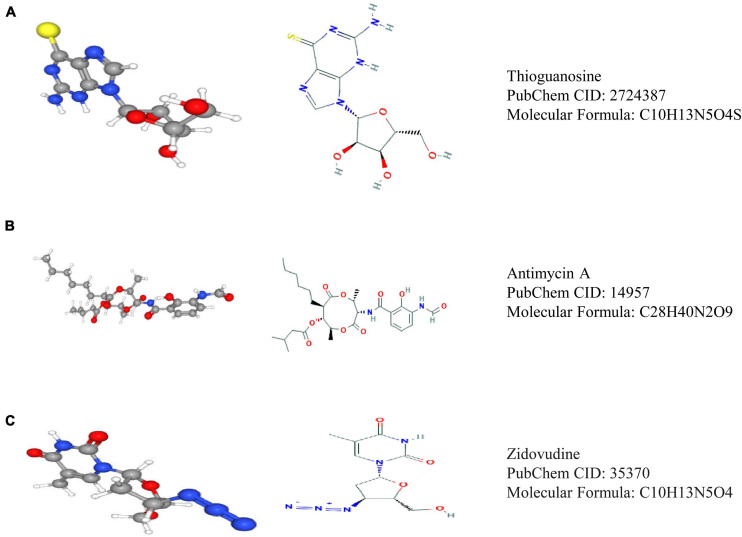
Three drugs predicted by CMap analysis results. **(A)** Structure diagram of thioguanosine, structure formula, and ID. **(B)** Structure diagram of antimycin A, structure formula, and ID. **(C)** Structure diagram of zidovudine, structure formula, and ID.

Through Pearson correlation analysis, we obtained 20 ESPL1-related genes using co-expression analysis. There were 10 genes (KIF2C, FAM64A, KIF20A, MKI67, ASPM, HJURP, KIF23, IQGAP3, TROAP, GTSE1) that were positively correlated and 10 (SPOCK2, LYNX1, CBX7, FBXW4, ADARB2, NEBL, MRVI1, SCN2B, ETNPPL, LDHD) that were negatively correlated (Figures 8A,B). We then uploaded these genes to CMap, which predicted three drugs that may harbor potential therapeutic effects on glioma: thioguanosine, antimycin A, and zidovudine (Table 3). The 2D and 3D structures of these small-molecule drugs are available from PubChem and are shown in Figure 9.

**TABLE 3:** Three small molecule compounds identified as potential drugs for glioma treatment in CMap analysis.

**CMap name**	**Mean**	***N***	**Enrichment**	***p*-Value**
Thioguanosine	−0.706	4	−0.944	0
Antimycin A	−0.592	5	−0.786	0.00084
Zidovudine	−0.586	4	−0.754	0.00734

*CMap, Connectivity Map.*

## Discussion

In the field of oncology, several studies have demonstrated that ESPL is related to the malignant biological behavior of many human tumors, promoting the development and proliferation of tumor cells and leading to poor outcomes. For example, Finetti et al. (2014) reported that ESPL1 is an oncogenic driver of luminal B breast cancers and has a powerful prognostic value. Furthermore, Hu et al. (2020) found that an HBV S-integrated human ESPL1 fusion gene may potentially represent a biomarker for the early diagnosis of HCC in HBV-infected patients. In addition, it has been reported that enhanced ESPL1 expression might be the reason for the increased malignancy of non-small cell and small cell lung cancer, and that ESPL1 represents a potential target for molecular therapy of lung cancer (Zhang et al., 2016). Similar conclusions have been verified in other malignant tumors, such as rectal adenocarcinoma, bladder cancer, and prostate carcinoma (Zhang and Pati, 2017; Chen et al., 2019). However, there is no literature on the relationship between ESPL1 and glioma. Elucidating the expression levels of ESPL1 in glioma and its clinical relevance will help to establish a new therapeutic target to improve existing treatment methods.

In this study, we first assessed the expression levels of ESPL1 in glioma using the GEPIA, GEO, and HPA databases. The results demonstrated that ESPL1 expression in glioma tissues was elevated compared to that in normal brain tissues at both the mRNA and protein levels. In addition, univariate and multivariate Cox analyses demonstrated that ESPL1 expression might be a useful biomarker for glioma prognosis and that ROC analysis confirmed the diagnostic value of ESPL1 expression in glioma. Moreover, Kaplan–Meier curves for OS showed that higher expression of ESPL1 was related to worse outcomes in glioma patients, especially in patients with low-grade gliomas. Furthermore, the potential mechanism of these results might be linked to homologous recombination, cell cycle, and base excision repair, as indicated by the GSEA results. These signaling pathways have been shown to play key roles in the biological behavior of many tumors in terms of metastasis and proliferation, indicating the potential role of ESPL1 as a new therapeutic and prognostic biomarker in glioma (Pennington et al., 2014; Wallace, 2014; Gavande et al., 2016; Ouyang et al., 2016; Christenson and Antonarakis, 2018). However, the function of this gene is realized in multiple ways. Therefore, further studies on the mechanism of ESPL1 in glioma are needed to clarify and expand upon these findings.

The abnormal expression of many genes is related to the pathological mechanism and malignant progression of glioma (Guan et al., 2018; Wang et al., 2018; Feng et al., 2020; Liu Z. et al., 2020). To determine whether ESPL1 associates with other genes to promote the malignant development of glioma, we further analyzed its co-expression and identified genes with co-expression relationships with ESPL1, confirming ESPL1 as a cancer gene with abnormally high expression that promotes the malignant progression of glioma. These results indicate that high expression of GTSE1, TROAP, IQGAP3, KIF23, HJURP, ASPM, MIKI67, KIF20A, FAM64A, and KIF2C might be unfavorable for glioma prognosis, whereas high expression of LDHD, ETNPPL, SCN2B, MRVI1, NEBL, ADARB2, FBXW4, CBX7, LYNX1, and SPOCK2 may be beneficial for the prognosis of glioma. For example, Sun et al. (2016) revealed that overexpression of KIF23 leads to unfavorable clinical outcomes in glioma and might be a useful independent prognostic biomarker for glioma patients (Sun et al., 2016). On the other hand, it has been reported that reduced expression of ETNPPL is closely related to the progression of glioma, particularly in glioblastoma (González-García et al., 2020). These results are consistent with our analysis. These genes also indirectly suggest that ESPL1 may promote the pathological processes of glioma and affect the prognosis of glioma patients.

Finally, the CMap database is an online platform for drug research and development. It can screen out drugs with potential therapeutic effects based on the change in gene expression level in the pathophysiological process of disease, so as to correct gene disorders and exert its therapeutic effects (Lamb et al., 2006). In this study, we screened three small-molecule drugs that may inhibit the occurrence and development of glioma through CMap analysis: thioguanosine, antimycin A, and zidovudine. The potential therapeutic effects of these small-molecule compounds on tumors have been described in the literature. For example, thioguanosine has been widely used in the treatment of acute leukemia (Evans and Relling, 1994). Subsequently, Kyritsis et al. (1996) demonstrated that chemotherapy with a combination of 6-thioguanine, procarbazine, lomustine, and hydroxyurea is effective for recurrent anaplastic gliomas (Kyritsis et al., 1996). In addition, as an antifungal drug, antimycin A was recently shown to inhibit the self-renewal ability of lung cancer stem cells by negatively regulating β-catenin signaling (Seipke et al., 2011; Yeh et al., 2013). Moreover, Wagner et al. (1997) revealed that zidovudine inhibits the activity of breast cancer. The above findings indicate that the small-molecule compounds inhibit tumor growth. However, these small-molecule drugs have not been reported to prevent anti-glioma cell proliferation. This study only provides an index to guide more researchers to pay attention to their potential value in the treatment of glioma. Drug repurposing can quickly understand the pharmacokinetics of drugs and evaluate their side effects, so that it can be applied to the first-line clinical practice (Ashburn and Thor, 2004; Boguski et al., 2009). For example, atorvastatin was considered to be a traditional classic antihyperlipidemic drug in the past, but it has been found to have a good therapeutic effect for chronic subdural hemorrhage in recent years (Jiang et al., 2018). Zidovudine has not been previously studied in the field of anti-glioma. However, it was later found that it can improve the sensitivity of glioma cells to radiotherapy, so as to play an anti-glioma therapeutic effect (Zhou et al., 2007). Therefore, these small-molecule drugs may have similar effects on glioma. Although there is no direct evidence that these compounds have an inhibitory effect on glioma, we have sufficient reason to support them as potentially effective drugs. However, additional studies are needed to examine their novel effects.

Although this study utilized multiple datasets with thousands of glioma samples for scientific analysis, revealing the mechanism of ESPL1 in glioma diagnosis and treatment will require additional studies. This study has several limitations. Since most of our data were from public databases, detailed treatment strategies were not available for each patient. However, it is precisely because of the multi-dataset fusion analysis that the statistical bias of race is reduced, which makes our results more reliable.

In summary, our results suggest that the overexpression of ESPL1 is closely related to poor prognosis in glioma patients. We believe that this study further improves our understanding of the pathogenesis of glioma and provides a novel and effective prognostic biomarker for gliomas, especially low-grade gliomas.

## Data Availability Statement

The original contributions presented in the study are included in the article/Supplementary Material, further inquiries can be directed to the corresponding author/s.

## Ethics Statement

The authors are accountable for all aspects of the work (including full data access, integrity of the data and the accuracy of the data analysis) in ensuring that questions related to the accuracy or integrity of any part of the work are appropriately investigated and resolved. Procedures of this work were approved by Ethics Committee of Affiliated of Henan Provincial People’s Hospital. The use of patient samples conformed to the declaration of Helsinki.

## Author Contributions

ZL, XL, YZ, and YG contributed to experimental design and implementation. XZ, XL, WZ, HW, and ZR performed the experiments. ZL, MZ, JW, and ML analyzed the data. XL and BL drafted the manuscript. All authors read and approved the final manuscript.

## Conflict of Interest

The authors declare that the research was conducted in the absence of any commercial or financial relationships that could be construed as a potential conflict of interest.

## Publisher’s Note

All claims expressed in this article are solely those of the authors and do not necessarily represent those of their affiliated organizations, or those of the publisher, the editors and the reviewers. Any product that may be evaluated in this article, or claim that may be made by its manufacturer, is not guaranteed or endorsed by the publisher.

## Footnotes

1
http://gepia.cancer-pku.cn/index.html

2
https://www.ncbi.nlm.nih.gov/geo/

3
http://www.cgga.org.cn/

4
https://portal.gdc.cancer.gov/

5
http://www.proteinatlas.org/

6
https://pubchem.ncbi.nlm.nih.gov/

7
https://www.proteinatlas.org/search/ESPL1
