# Supplementary material for: ESPL1 Is a Novel Prognostic Biomarker Associated With the Malignant Features of Glioma
Source: Front Genet. 2021 Aug 26;12:666106. doi: 10.3389/fgene.2021.666106 (PMC8428966; doi:10.3389/fgene.2021.666106)
Supplement: Supplementary file 1 [file Data_Sheet_1.docx]

**Figure S1**


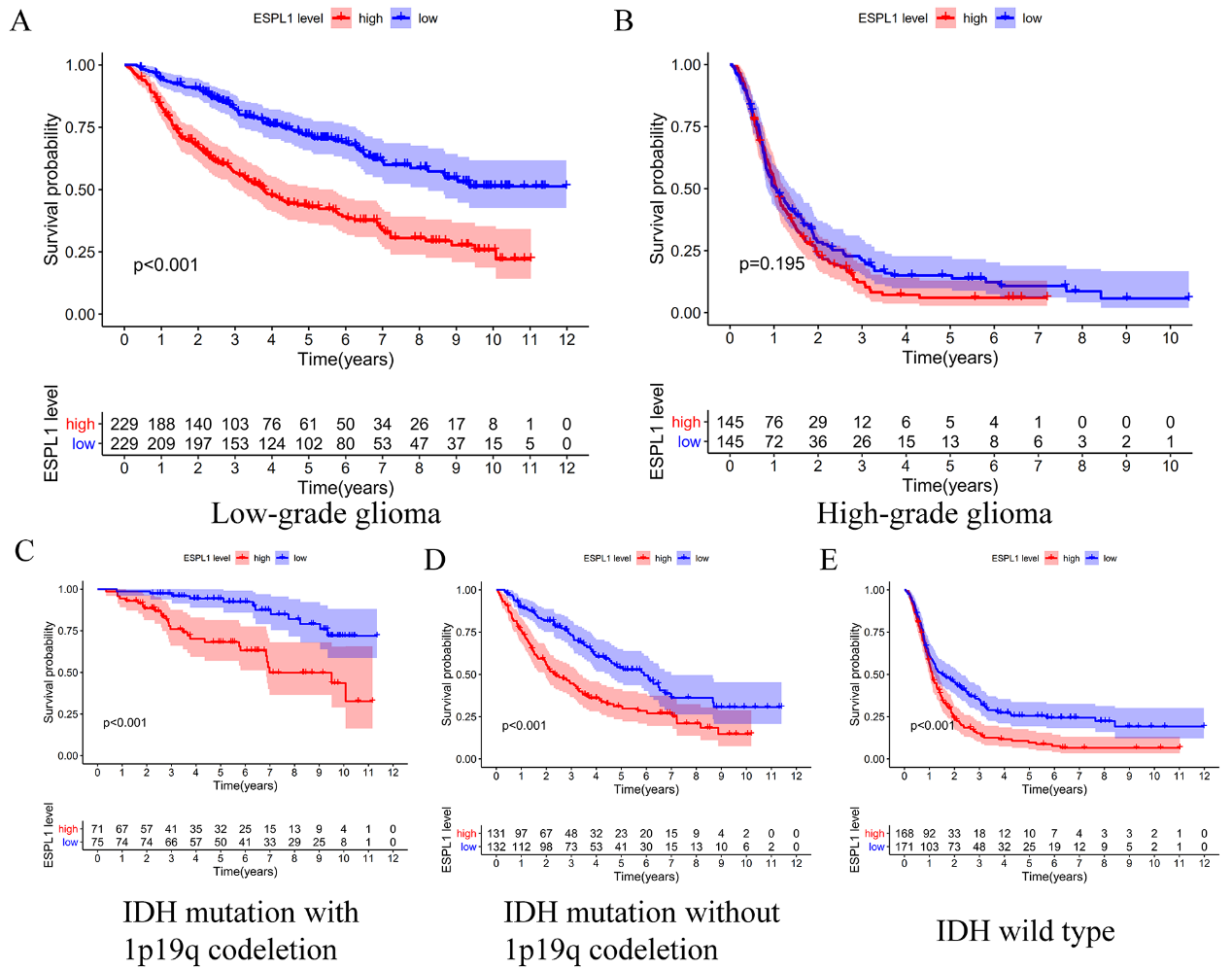
Figure S1: The relationship between the difference of the expression level of ESPL1 and Overall Survival (OS) in CGGA sequence. The data is highly expressed and low expression according to the median value of ESPL1 expression. Red represents high expression ESPL1, blue represents low expression ESPL1. (A) Effects of ESPL1 expression difference on patient survival in low-level gliomas. (B) Effects of ESPL1 expression difference on patient survival in high-grade glioma. (C-E) The effect of ESPL1 expression between different molecular categories on patient survival, including IDH mutation with 1p19q codeletion, IDH mutation without 1p19q codeletion, IDH Wild Type.

**Figure S2**


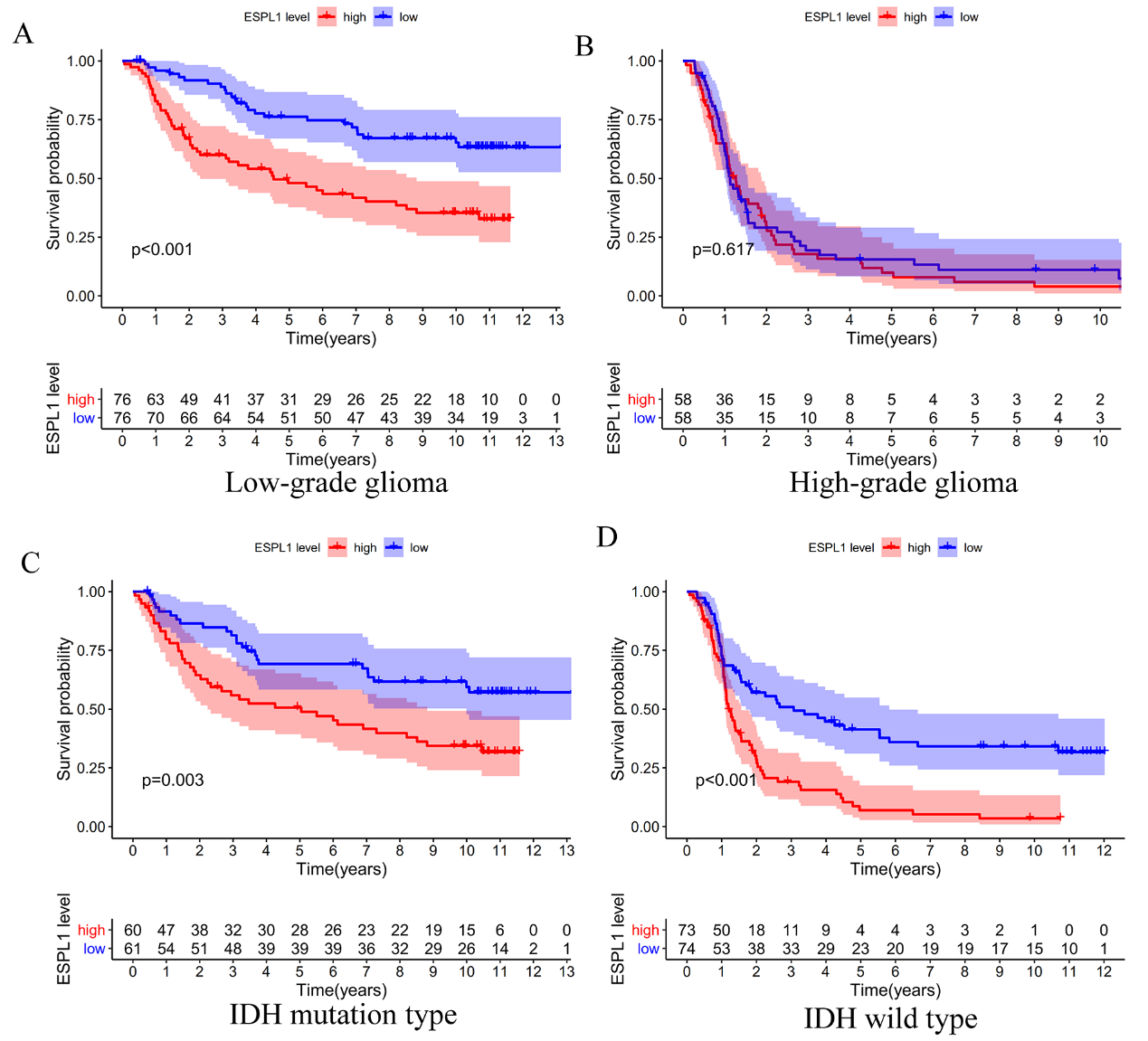


Figure S2: The relationship between the difference of the expression level of ESPL1 and Overall Survival (OS) in CGGA microarray. (A) Effects of ESPL1 expression difference on patient survival in low-level gliomas. (B) Effects of ESPL1 expression difference on patient survival in high-grade glioma. (C-D) Effects of ESPL1 expression difference on patient survival under IDH mutation type and IDH wild type.

**Figure S3**


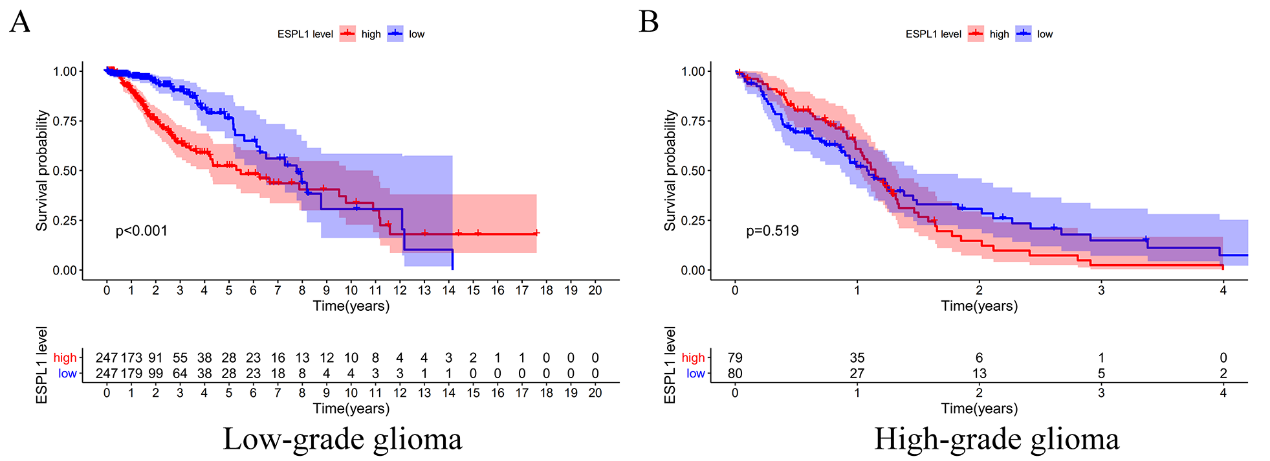


Figure S3: The relationship between the difference of the expression level of ESPL1 and Overall Survival (OS) in TCGA RNA-Seq. (A) Effects of ESPL1 expression difference on patient survival in low-level gliomas. (B) Effects of ESPL1 expression difference on patient survival in high-grade glioma.

**Figure S4**


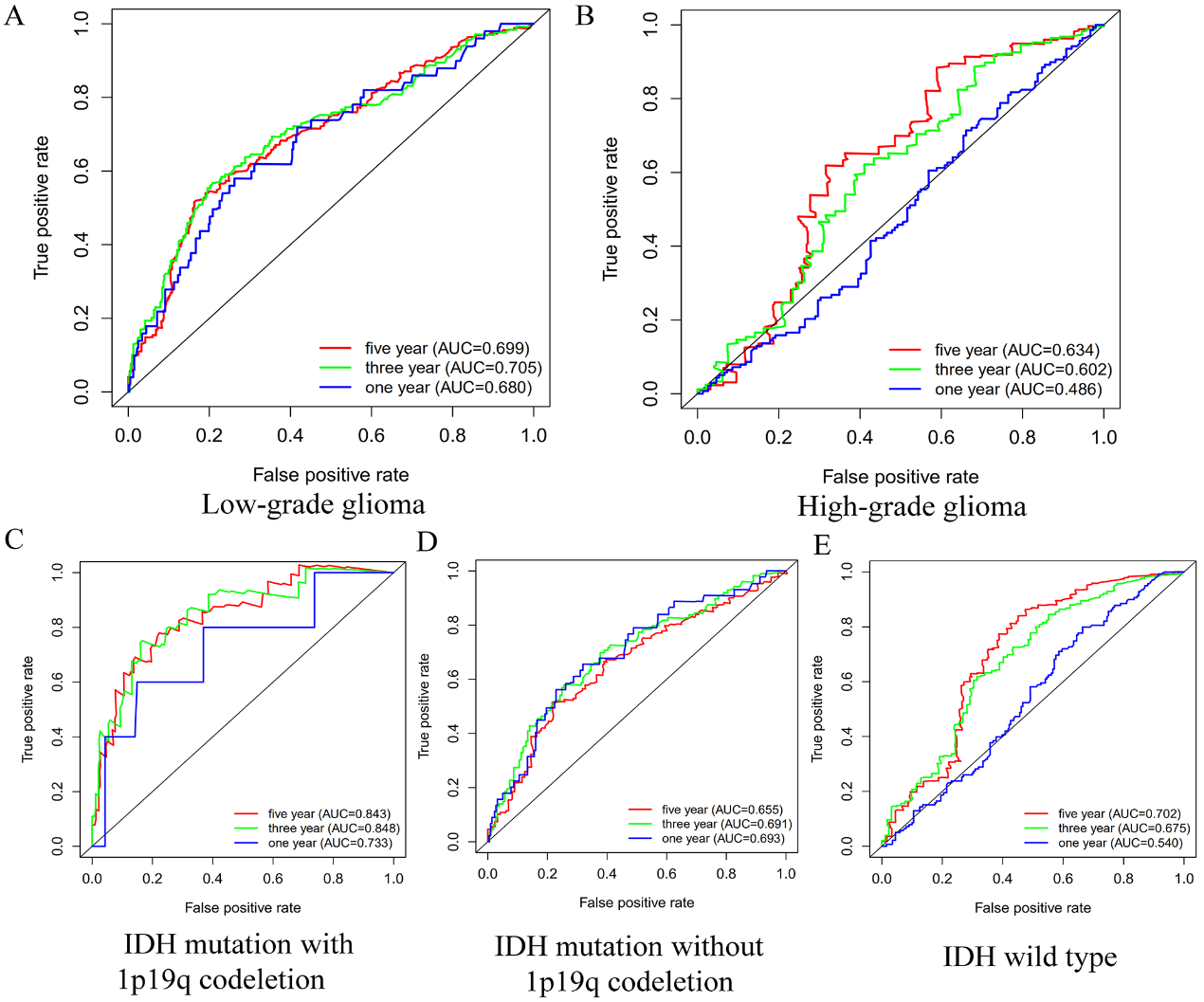


Figure S4: Prognostic factors and risk assessment of glioma and the diagnostic value of ESPL1 in CGGA sequence. (A) The receiver operating characteristic (ROC) curve of low-grade glioma. (B) ROC curve of high-grade glioma. (C) ROC curve of IDH mutation with 1p19q codeletion. (D) ROC curve of IDH mutation without 1p19q codeletion. (E) ROC curve of IDH wild type.

**Figure S5**


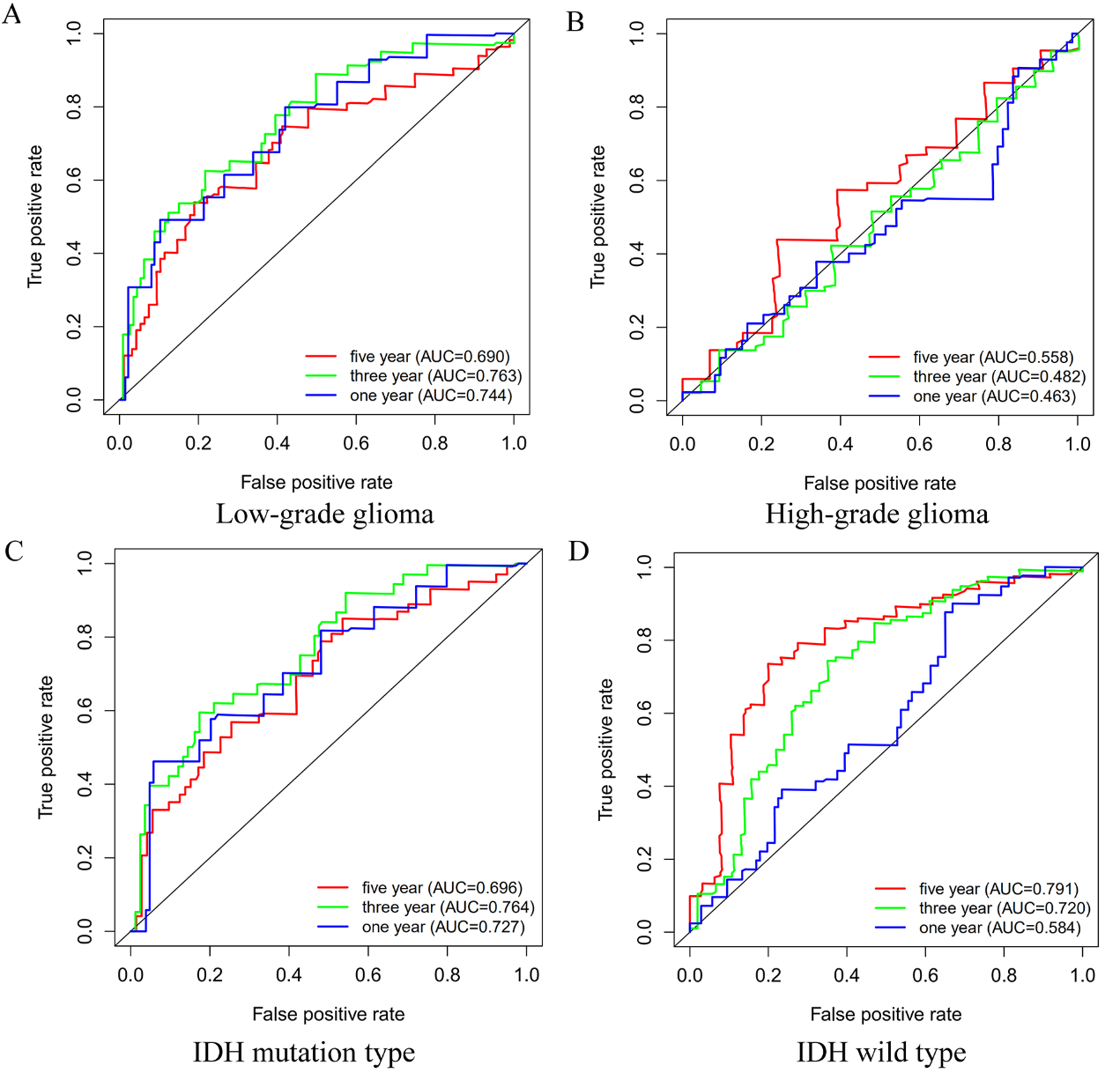


Figure S5: Prognostic factors and risk assessment of glioma and the diagnostic value of ESPL1 in CGGA microarray. (A) The receiver operating characteristic (ROC) curve of low-grade glioma. (B) ROC curve of high-grade glioma. (C) ROC curve of IDH mutation type. (D) ROC curve of IDH wild type.

**Figure S6**


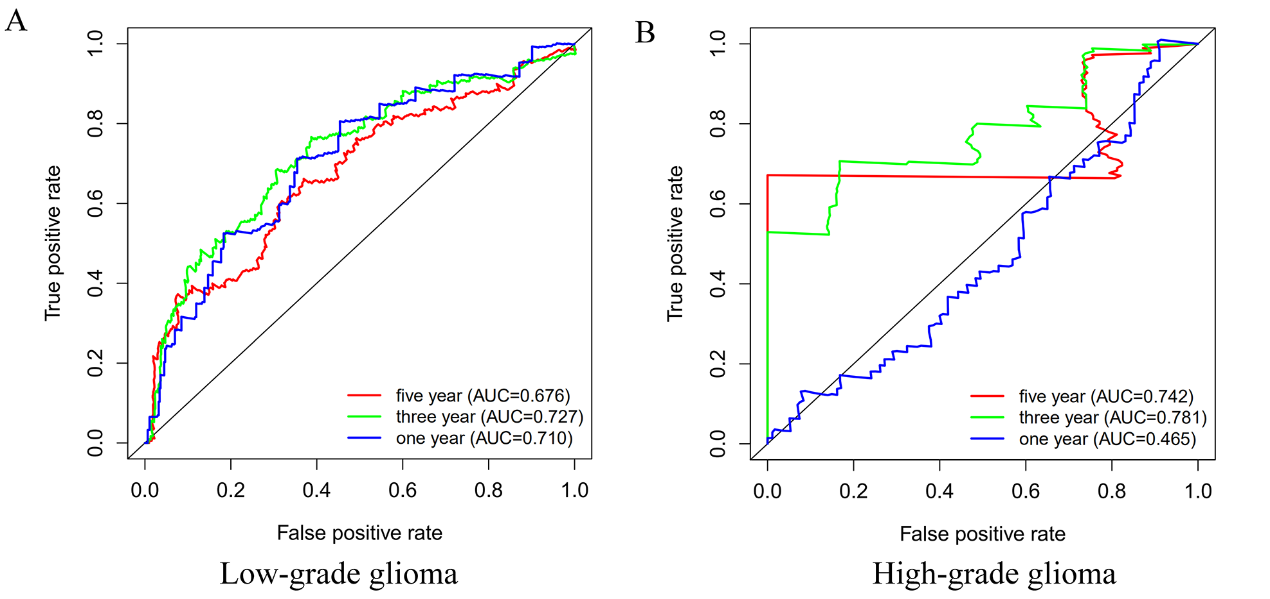


Figure S6: Prognostic factors and risk assessment of glioma and the diagnostic value of ESPL1 in TCGA RNA-Seq. (A) The receiver operating characteristic (ROC) curve of low-grade glioma. (B) ROC curve of high-grade glioma.

**Table S1.** Characteristics of patients with glioma based on CGGA RNA-seq data.

| Characteristics |  | Number of cases | Percentages (%) |
| --- | --- | --- | --- |
| Gender | Male | 306 | 40.91 |
|  | Female | 442 | 59.09 |
| Age | <=41 | 341 | 45.59 |
|  | >41 | 407 | 54.41 |
| Grade | WHO II | 218 | 29.14 |
|  | WHO III | 240 | 32.09 |
|  | WHO IV | 290 | 38.77 |
| PRS_type | Primary | 501 | 66.98 |
|  | Recurrent | 222 | 29.68 |
|  | Secondary | 25 | 3.34 |
| Radio_status | Yes | 625 | 83.56 |
|  | No | 123 | 16.44 |
| Chemo_status | Yes | 520 | 69.52 |
|  | No | 228 | 30.48 |
| Histology | Astrocytoma | 55 | 7.35 |
|  | Anaplastic astrocytoma | 39 | 5.21 |
|  | Anaplastic oligodendroglioma | 22 | 2.94 |
|  | Anaplastic oligoastrocytoma | 80 | 10.70 |
|  | Glioblastoma | 175 | 23.40 |
|  | Oligodendroglioma | 35 | 4.68 |
|  | Oligoastrocytoma | 95 | 12.70 |
|  | Relapse astrocytoma | 20 | 2.67 |
|  | Relapse anaplastic astrocytoma | 36 | 4.81 |
|  | Relapse anaplastic oligodendroglioma | 15 | 2.01 |
|  | Relapse anaplastic oligoastrocytoma | 48 | 6.42 |
|  | Relapse glioblastoma | 90 | 12.03 |
|  | Relapse oligodendroglioma | 4 | 0.53 |
|  | Relapse oligoastrocytoma | 9 | 1.20 |
|  | Secondary relapse glioblastoma | 25 | 3.34 |
| IDH_mutation_status | Mutant | 409 | 54.68 |
|  | Wildtype | 339 | 45.32 |
| 1p19q_codeletion_status | Codel | 155 | 20.72 |
|  | Non-codel | 593 | 79.28 |

**Table S2.** Characteristics of patients with glioma based on CGGA microarray data.

| Characteristics |  | Number of cases | Percentages(%) |
| --- | --- | --- | --- |
| Gender | Male | 153 | 57.09 |
|  | Female | 115 | 42.91 |
| Age | <=42 | 138 | 51.49 |
|  | >42 | 130 | 48.51 |
| Grade | WHO II | 100 | 37.31 |
|  | WHO III | 52 | 19.40 |
|  | WHO IV | 116 | 43.28 |
| PRS_type | Primary | 238 | 88.81 |
|  | Recurrent | 20 | 7.46 |
|  | Secondary | 10 | 3.73 |
| Radio_status | Yes | 240 | 89.55 |
|  | No | 28 | 10.45 |
| Chemo_status | Yes | 145 | 54.10 |
|  | No | 123 | 45.90 |
| Histology | Astrocytoma | 63 | 23.51 |
|  | Anaplastic astrocytoma | 25 | 9.33 |
|  | Anaplastic oligodendroglioma | 10 | 3.73 |
|  | Anaplastic oligoastrocytoma | 4 | 1.49 |
|  | Glioblastoma | 102 | 38.06 |
|  | Oligodendroglioma | 21 | 7.84 |
|  | Oligoastrocytoma | 13 | 4.85 |
|  | Relapse astrocytoma | 3 | 1.12 |
|  | Relapse anaplastic astrocytoma | 9 | 3.36 |
|  | Relapse anaplastic oligodendroglioma | 4 | 1.49 |
|  | Relapse glioblastoma | 4 | 1.49 |
|  | Secondary relapse glioblastoma | 10 | 3.73 |
| IDH_mutation_status | Mutant | 121 | 45.15 |
|  | Wildtype | 147 | 54.85 |

**Table S3.** Characteristics of patients with glioma based on TCGA RNA-seq data.

| Characteristics |  | Number of cases | Percentages(%) |
| --- | --- | --- | --- |
| Gender | Male | 377 | 57.73 |
|  | Female | 276 | 42.27 |
| Age | <=51 | 394 | 60.34 |
|  | >51 | 259 | 39.66 |
| Grade | WHO II | 238 | 36.45 |
|  | WHO III | 256 | 39.20 |
|  | WHO IV | 159 | 24.35 |
